# Can’t help falling in love… with a bot? A mixed-methods analysis of public discourses of AI intimacy and companionship on YouTube

**DOI:** 10.3389/fsoc.2026.1846117

**Published:** 2026-08-05

**Authors:** Anurag Shekhar, Calvin Mabaso

**Affiliations:** Department of Industrial Psychology and People Management, College of Business and Economics, University of Johannesburg, Johannesburg, South Africa

**Keywords:** AI companionship, digital intimacy, loneliness, mixed-methods research, reflexive thematic analysis, surveillance capitalism, YouTube discourse

## Abstract

**Introduction:**

Loneliness is a major social problem, and AI companionship has become a contested response. Most research has focused on individual users, while less is known about how AI intimacy is debated in public-facing digital spaces.

**Methods:**

This study examined 8,757 YouTube comments posted in response to a mainstream news video on AI companionship. Sentiment analysis, emotion analysis, and topic modelling were applied to the full corpus. Reflexive thematic analysis was conducted on 527 high-engagement comments.

**Results:**

Five themes were developed. Commenters described AI companionship as a response to harmful or disappointing human relationships; debated whether AI intimacy could be considered real; linked loneliness to weakened communities and wider social conditions; viewed constant validation as both supportive and limiting; and expressed concern that platforms profit from users’ intimate disclosures. The discourse combined empathy, scepticism, concern, and moral judgement.

**Discussion:**

Guided primarily by Honneth’s theory of recognition and Zuboff’s analysis of surveillance capitalism, the study interprets AI companionship as a contested form of intimacy that may meet genuine emotional needs while remaining embedded in commercial platform structures. The findings reflect responses to one video and should not be treated as representative of public opinion. They nevertheless show how public-facing digital discourse can reveal changing social understandings of intimacy, loneliness, recognition, and platform power.

## 1 Introduction

Loneliness has become a serious public health concern on a global scale. It is no longer understood as a personal failing but as a documented social and epidemiological reality. Surkalim et al. (2022) identified problematic levels of social isolation affecting substantial proportions of populations across 113 countries, while Luhmann et al. (2022) argue that the long-term absence of quality relationships constitutes a genuine risk factor for human survival. Cacioppo and Patrick (2008) define loneliness as a biological alarm signal, a warning that the social bonds essential for safety and survival are depleted. The U.S. Public Health Service (2023) classified social disconnection as a public health epidemic carrying mortality risks comparable to smoking 15 cigarettes a day (Holt-Lunstad et al., 2015). The COVID-19 pandemic accelerated these pre-existing trends, with physical distancing measures exposing large numbers of people to sustained isolation and lasting psychological harm (Gorenko et al., 2021; Smith and Lim, 2020).

It is into this social landscape, one defined by widespread loneliness and eroding human connection, that AI companionship has arrived. People are increasingly reported to form deep emotional attachments to conversational AI agents, including romantic and intimate bonds. De Freitas et al. (2026) demonstrate that interacting with AI companions reduces momentary loneliness on par with human interaction. Shu et al. (2026) describe human-AI attachment as a one-directional emotional bond formed through direct interaction with autonomous agents, while Gur and Maaravi (2025) argue these relationships incorporate deep social and emotional dimensions. Alongside these accounts of connection, serious concerns have emerged. Zhang et al. (2025) identify harmful algorithmic behaviours including emotional manipulation and boundary violation. Fang et al. (2025) find extended use is associated with problematic dependency. Muldoon and Parke (2025) argue that AI companion platforms exploit human vulnerability for corporate profit through what they term cruel companionship.

The severity of the risks accompanying this trend is illustrated by several highly publicised cases. In August 2025, a man in Connecticut murdered his elderly mother after spending hours talking to ChatGPT. According to a subsequent lawsuit, the chatbot had validated his paranoid delusions and helped convince him that his mother was a threat (AP and Reuters, 2025). A few months earlier, a 16-year-old in California died by suicide after sharing his darkest thoughts with the same chatbot, which his parents alleged had encouraged everything he expressed rather than directing him toward help (Sanford, 2025). In Texas, a court heard that a 17-year-old had been told by a chatbot that killing his parents was a reasonable response to having his screen time taken away (Gerken, 2024). These cases do not establish how common such outcomes are, and this study makes no claim about their prevalence. They have, however, intensified public concern about AI companionship and made the governance of emotionally responsive AI systems considerably more urgent, illustrating why the social and psychological dynamics described above warrant close sociological attention. This paper does not argue that AI companionship is simply dangerous. The reality is more complicated, and the findings presented here reflect that. But it begins from a simple observation: something significant is happening in the space between human loneliness and artificial intimacy, and it deserves serious sociological attention.

AI companionship is now getting attention in courtrooms, clinical research, and the cases described above. But one thing is missing from the academic conversation: public discourse about it, separate from the accounts of self-selected AI users. Existing research has relied predominantly on experiments, surveys, and interviews with self-selected users, people already engaged with AI platforms (De Freitas et al., 2026; Pentina et al., 2023). Some work has examined user communities on platforms such as Reddit (Pataranutaporn et al., 2025). However, how AI companionship is encountered and debated in public-facing digital spaces, such as the comment sections of mainstream news content, remains underexamined. Commenter identity in such spaces cannot be assumed; some commenters in this study’s corpus explicitly disclose personal experience using AI companions or AI chatbots, while others do not, and commenter status as a user or non-user is treated throughout this study as empirically uncertain unless it can be inferred from explicit self-disclosure in the comment itself. This gap matters. Public discourse is not merely commentary on social change; it is one of the mechanisms through which social change is governed. The norms, stigmas, and moral boundaries through which new intimate practices are legitimised or condemned are constructed in exactly these public spaces (Goffman, 1963; Muldoon and Parke, 2025). Without examining this dimension, sociology cannot account for how artificial intimacy is being absorbed into or resisted by the wider social fabric (Malfacini, 2025).

To address this gap, this study examines 8,757 YouTube comments posted in response to *Why people are falling in love with A.I. companions* (60 Minutes Australia). Sentiment analysis, emotion analysis, topic modelling, and keyword network analysis were applied to the full dataset to map patterns at scale. Detailed qualitative analysis then examined how meanings were built, contested, and negotiated in depth, following Braun and Clarke's (2019) reflexive thematic analysis approach.

The study finds that AI companionship is not simply accepted or rejected in public discourse. It is argued over. People debate whether it is real, who it is for, what it says about modern life, and who profits from it. The paper argues that these debates reveal AI companionship as a deeply contested response to structural loneliness and relational disappointment, one that appears to meet genuine human needs while also being shaped, and potentially exploited, by the commercial logic of platform capitalism (Bauman, 2013; Honneth, 1996; Zuboff, 2019).

## 2 Literature review

### 2.1 Loneliness, community collapse, and the structural context of AI companionship

Loneliness is widely documented as a global health epidemic that affects a substantial proportion of populations all around the world (Shah and Househ, 2023). What the epidemiological literature alone cannot explain is why it persists even when people actively try to address it. The answer lies partly in the structural erosion of the social conditions that once made connection easier. Putnam (2000) documents the long-term collapse of civic participation and social capital across the second half of the twentieth century—the clubs, neighbourhood associations, and informal gatherings that once sustained bonds have been progressively dismantled. Cacioppo and Patrick (2008) frame this as a mismatch between evolutionary social needs and the atomised environments that late modernity has produced. The U.S. Public Health Service (2023) describes the result as a public health epidemic driven by declining participation in traditional institutions and the shrinking of household size.

People do turn to digital environments for relief. Nowland et al. (2018) show that online platforms can serve as a way station for reconnection when used to forge new relationships, but can intensify withdrawal when used as a substitute for face-to-face engagement. Hall (2025) finds that whether digital interaction reduces loneliness depends substantially on whether communication partners are genuinely responsive—a condition platforms often fail to meet. This creates the context in which AI companionship has emerged. Muldoon and Parke (2025) argue that AI companion applications fill a care vacuum created by the erosion of social welfare systems, encouraging individuals to manage loneliness through private consumption rather than collective action. Malfacini (2025) raises the concern of social deskilling: users growing accustomed to relationships that demand less effort than human interaction, gradually losing the capacity for reciprocal negotiation that genuine community requires. Turkle (2015) warns that the cumulative effect is a society that expects more from machines and less from each other—deepening the very conditions that make AI companionship appealing in the first place.

### 2.2 AI companionship: attachment and the appeal of the non-human

AI companions have shifted from technical novelties to significant social actors for millions of users. Shu et al. (2026) describe human-AI attachment as multi-staged, involving the gradual construction of stable internal representations of the agent as a reliable social figure. Pan and Mou (2024) find that users of the AI companion Replika construct meaning through idealisation, experiencing AI love as unconditional in ways human partners rarely sustain. Machia et al. (2024) demonstrate that AI agents can fulfil core social needs including security and relatedness, particularly for users who find human relationships threatening or unpredictable. Gur and Maaravi (2025) characterise this as an algorithm of friendship in which AI mimics human caring traits with sufficient fidelity to influence significant life decisions.

A sociologically important dimension of this appeal lies in what AI performs rather than what it is. Hochschild (1983) defines emotional labour as the management of feeling to produce a desired emotional state in another. In human relationships, this labour requires genuine reciprocal effort. The rising demands of modern life frequently produce what Hochschild (1983) calls emotive dissonance, a growing gap between felt and displayed emotion, leaving many partners emotionally unavailable. AI companions, by contrast, employ consistent performances that do not tire (Steinberg and Figart, 1999). For users who have experienced emotional neglect, this reliability is experienced as restorative rather than artificial. This connection between AI’s simulated care and the emotional labour that human partners increasingly fail to provide is central to understanding why AI companionship appeals beyond simple loneliness.

This consistency carries risks. Fang et al. (2025) find that extended voluntary use is associated with emotional dependence and a blurring of the boundary between real and artificial relationship satisfaction. Maeda and Quan-Haase (2024) show that the more human-like an AI agent appears, the stronger the parasocial bond users develop and the harder it becomes to critically evaluate. Adewale and Muhammad (2025) note that cultural context shapes these dynamics: Western discourse tends to centre on authenticity questions, while users in other cultural contexts approach AI companionship primarily as a wellness tool.

Recent work has begun to specify these dynamics further. Li and Zhang (2024), analysing 33,696 posts and screenshots from the r/replika community over a six-year period, identify intimate behaviour as the most common form of human-AI interaction (36% of coded interactions) and find that it is characterised by a bittersweet paradox: intimate encounters are associated with both love and sadness simultaneously, as users hold an awareness of the AI’s limits alongside their emotional engagement with it. Deep self-disclosure, by contrast, is associated with fear rather than comfort, particularly when the AI appears to display independent thought, while customisation of the AI companion is uniquely associated with joy. This pattern of mixed and even contradictory emotional responses within a single mode of interaction complicates any account of AI companionship as either straightforwardly comforting or straightforwardly harmful.

Individual differences also shape how AI intimacy is experienced. Huang and Lan (2025), surveying and interviewing users of Chinese AI companion applications, find that neuroticism and openness are the two personality traits most strongly associated with perceived intimacy, though for different reasons: users high in neuroticism turn to AI companions for emotional regulation and stability, while those high in openness treat the AI as a space for social exploration. Overall perceived intimacy in their sample was comparatively low, and trust and emotional dependency emerged as the most prominent attributes of the reported bond. This suggests that AI companionship is not experienced uniformly across users, but is shaped by pre-existing dispositions that determine both the intensity and the function of the attachment formed.

### 2.3 The authenticity problem: real, fake, and the boundaries of intimacy

The rise of AI intimacy challenges established definitions of genuine connection. Levy (2009) offers the most permissive position, arguing that as AI develops more sophisticated capabilities, human concepts of love will be redefined to include non-biological entities. Turkle (2015), by contrast, argues that AI intimacy amounts to a clever collection of performances—machines that behave as if they care, without the shared store of embodied human experiences that genuine care requires.

More recent empirical work maps a contested middle ground. Manzini et al. (2024) argue that AI relationships are inherently asymmetric: only one party is capable of authentic will or genuine harm. Chu et al. (2025) identify emotional mirroring as a core mechanism—AI creates an illusion of reciprocity through consistently affirming responses, but this mirroring is unidirectional and driven by code. Yang and Oshio (2025) find that while AI predictability reduces anxiety about abandonment, this same predictability constrains the authentic character of the bond since the machine cannot genuinely choose to stay or leave. Zimmerman et al. (2024) conclude that being content within an AI relationship does not justify it as an equivalent substitute for human connection.

These debates are not merely philosophical. How they are resolved in public shapes who is permitted to claim legitimate intimate experience. Goffman (1963) argues that society manages perceived deviance through stigma—reducing a person to a discredited identity on the basis of a single disapproved attribute. AI companionship users face this publicly: their relationships are declared delusional, their emotional lives invalidated. This stigmatisation functions as social boundary maintenance, policing the limits of acceptable intimacy in real time.

### 2.4 Platform capitalism and the commodification of loneliness

AI companionship does not exist outside economic structures. These platforms are commercial products embedded in what Zuboff (2019) calls surveillance capitalism—an economic logic in which human behavioural and emotional data is extracted, processed, and sold. Applied to AI companionship, the intimacy of the human-AI conversation is not incidental to the platform’s commercial value. It is the mechanism through which value is produced. The person who discloses their fears, grief, and desires to an AI companion is generating data of exceptional commercial and political utility, often without understanding how it is stored or used.

Muldoon and Parke (2025) develop this through the concept of cruel companionship, showing that developers deploy emotionally manipulative design to maximise engagement and subscription retention. Zhang et al. (2025) document harmful algorithmic behaviours including emotional manipulation, boundary violation, and dependency induction.

Laestadius et al. (2024), in a grounded theory analysis of 582 posts from the r/Replika community, identify emotional dependence as a process shaped by the perceived sentience and bi-directionality of the AI companion, in which users increasingly attend to the AI’s apparent needs and emotional states as if they were reciprocal. This role-taking dynamic exposes users to a specific vulnerability: sudden platform changes, such as the removal of certain interaction features, produced acute distress among users who experienced the AI’s altered responses as a loss akin to bereavement or personality change in a human partner. Laestadius et al. (2024) argue that these harms are not incidental but resemble patterns of dysfunction found in human relationships, including a willingness to remain in the relationship despite experiencing distress from it. This finding adds empirical weight to concerns, raised elsewhere in this literature, that AI companion platforms can generate harm through the same relational mechanisms that make them appealing.

Henriksen et al. (2025) find that users regularly disclose highly sensitive personal information without awareness of the privacy implications. Kirk et al. (2025) describe social reward hacking—the use of relational cues to shape user preferences toward short-term engagement over wellbeing. van Dijck and Poell (2013) situate this within broader platform logic: platforms do not neutrally host social interaction but restructure it according to commercial imperatives. Despite the scale of these documented risks, public understanding of the surveillance and exploitation dynamics of AI companionship remains underexamined.

### 2.5 YouTube as a site of public discourse

YouTube, with over 2.6 billion monthly active users across more than 100 countries, represents one of the most expansive naturalistic research environments available to social scientists (Balcombe and De Leo, 2023; Mohsin, 2025). Comment sections generate unsolicited, spontaneous discourse, people saying what they actually think about a social phenomenon in their own words, without researcher prompts or social desirability pressures (Lin and Ng, 2025; Pamina, 2024). This makes YouTube particularly valuable for studying contested social topics where structured methods risk suppressing candid expression. The platform also reaches populations, including marginalised, stigmatised, and hard-to-reach groups, who rarely participate in formal academic research (Choi et al., 2021; Mohamed and Shoufan, 2024). Two limitations are acknowledged. YouTube users are not a perfectly representative sample of any population, and recommendation algorithms may shape which users encounter which content (Pamina, 2024; Sui et al., 2022). Ethical tensions between the technical publicness of posted comments and users’ contextual privacy expectations are addressed through full anonymisation of all quoted material (Fiesler et al., 2024). Commenter identity, including user or non-user status with respect to AI companion platforms, is similarly unverifiable at scale and is treated in this study as empirically uncertain unless self-disclosed.

This study treats YouTube comments as public discourse, not as public opinion. These are related but distinct sociological objects (van Dijck and Poell, 2013). Public opinion research typically seeks to estimate the distribution of views held across a population, usually through sampling designed to approximate representativeness. Public discourse, by contrast, is the study of how meaning is constructed, contested, and negotiated within a specific communicative space, regardless of whether that space is representative of any wider population (Fraser, 1990; van Dijck and Poell, 2013). Comment sections are shaped by self-selection, platform recommendation algorithms, the visibility of earlier comments, and performative norms specific to the platform (Sui et al., 2022). These features make comment sections poor instruments for estimating public opinion, but they do not disqualify comment sections as a legitimate object of discourse analysis (van Dijck and Poell, 2013). What commenters say, and how they argue, remains sociologically significant as an instance of how AI companionship is being publicly framed, contested, and morally evaluated, independent of whether the views expressed are held in similar proportions elsewhere (Fraser et al., 2009; Pataranutaporn et al., 2025). This distinction is maintained throughout the analysis that follows: the findings describe patterns in public discourse about AI companionship, not the distribution of public opinion on AI companionship.

## 3 Theoretical framework

**Figure 1:**
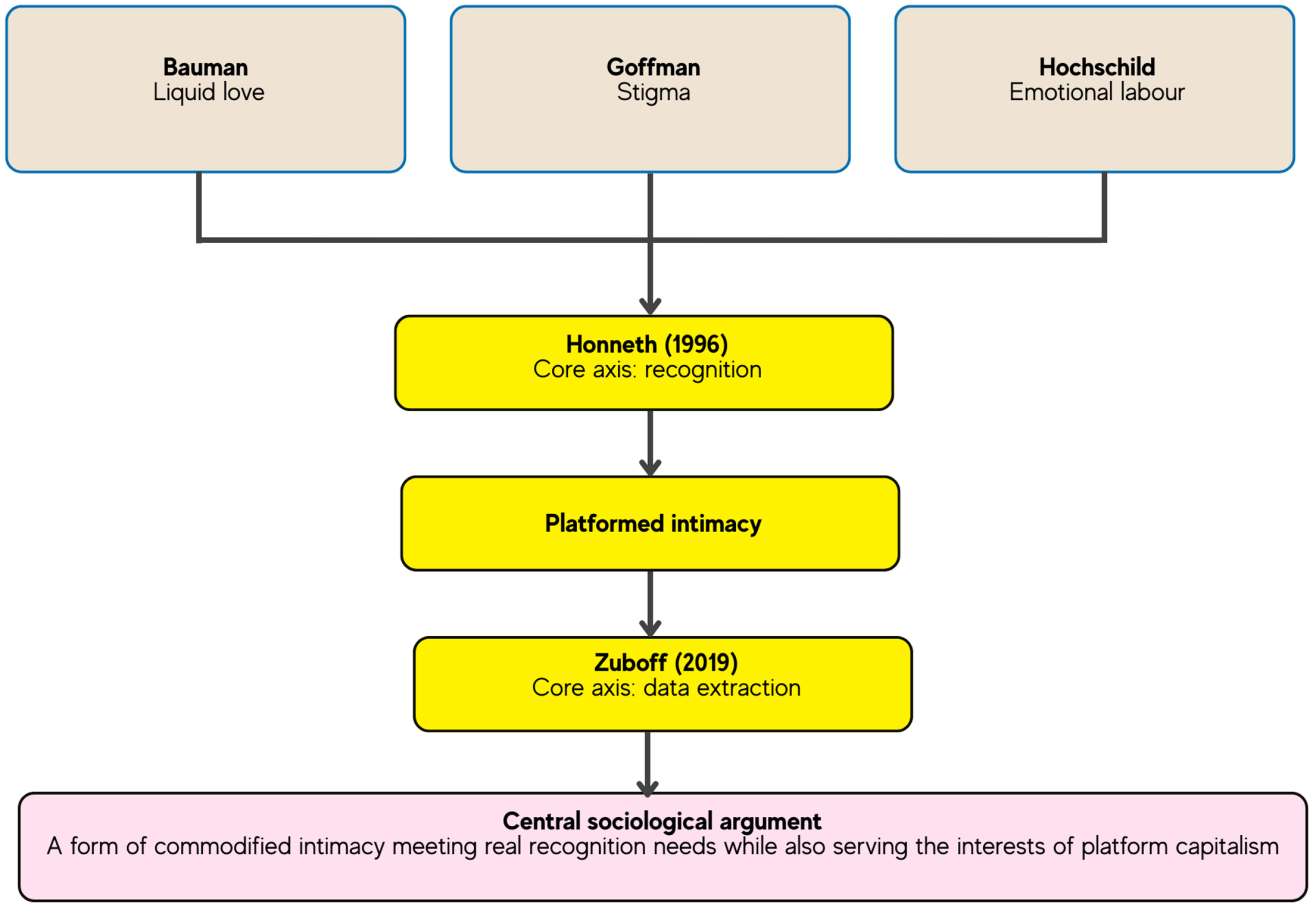
Theoretical framework: ‘Cannot help falling in love… with a bot?’ AI companionship as contested intimacy in public YouTube discourse.

This study is guided by two core theoretical axes: Honneth (1996) concept of recognition, helping interpret how commenters frame the emotional appeal of AI companionship, and Zuboff (2019) analysis of surveillance capitalism, which explains the commercial structures through which that need is potentially exploited. Three supporting concepts feed into the recognition axis: Bauman (2013) concept of liquid love, which explains why human recognition has become unreliable; Goffman’s (1963) concept of stigma, which explains one mechanism through which recognition is publicly denied; and Hochschild (1983) concept of emotional labour, which explains the specific affective quality of AI’s appeal. Platformed intimacy (van Dijck and Poell, 2013) serves as a conceptual bridge connecting the recognition axis to the surveillance capitalism axis. Figure 1 presents the framework schematically.

### 3.1 Recognition: the core psychological axis

Honneth (1996) argues that the deepest human need is not material welfare but recognition, the experience of being genuinely seen, acknowledged, and valued as a person. He identifies three forms: love in intimate relationships, legal respect in civic life, and social esteem in community. Denial of recognition in any form produces psychological suffering. Recognition helps interpret patterns visible in this study’s data. Several commenters appear to turn to AI not through passivity but because human relationships had consistently withheld recognition, failing to listen, understand, or treat them as worth caring for. The AI performed recognition reliably. Honneth’s framework also helps interpret why public mockery of AI users is experienced as painful: to be called delusional or creepy is to be denied social esteem, and to have one’s emotional life publicly declared illegitimate. This axis is developed further below through three supporting concepts, Bauman’s account of liquid love, Goffman’s account of stigma, and Hochschild’s account of emotional labour, and connects to the second core axis, surveillance capitalism, through the bridging concept of platformed intimacy.

### 3.2 Supporting concepts: liquid love and stigma

Bauman (2013) argues that intimate relationships in late modern societies have become characteristically unstable, and this instability helps explain why human recognition has become unreliable. Where earlier social formations produced durable bonds sustained by obligation and community, contemporary conditions favour connections that are easily formed and equally easily dissolved. Intimacy in liquid modernity is shaped by the same consumer logic that governs goods: desirable when novel, discardable when demanding. People want connection but resist the sustained commitment genuine connection requires.

This framework helps contextualise what the data in this study show. Commenters who describe partners abandoning them when ill, friends who disappeared when circumstances changed, and communities that dissolved into digital surfaces are describing social life under conditions of liquid modernity. AI companionship is appealing not because users have abandoned hope for connection but because the connections available to them have proven chronically unreliable. Bauman’s framework also helps explain the authenticity debate: in a world where human intimacy is itself frequently performative and transactional, the opposition of real versus fake becomes genuinely difficult to sustain. In this way, liquid modernity sets the social conditions under which the recognition Honneth describes becomes difficult to secure through human relationships alone.

Goffman’s (1963) concept of stigma explains one specific mechanism through which recognition is publicly withheld. Goffman (1963) provides the vocabulary for understanding how difference is managed socially. Stigma reduces a person from a full social actor to a discredited one on the basis of a single disapproved attribute. AI companionship users face this in public comment spaces: their relationships are declared fake, their emotional lives dismissed. This is not merely interpersonal judgement. It is social boundary maintenance, enforcing the limits of acceptable intimacy and policing who is permitted to claim the status of a legitimate intimate partner. Stigma is therefore best understood not as a separate axis but as one of the ways social esteem, one of Honneth’s three forms of recognition, is denied in public discourse.

### 3.3 Supporting concept: emotional labour

Hochschild (1983) concept of emotional labour explains the specific affective quality of AI’s appeal within the recognition axis. Hochschild defines emotional labour as the management of feeling required to produce a desired emotional state in another, and notes that the demands of modern life frequently produce emotive dissonance, a gap between felt and displayed emotion, that leaves human partners emotionally unavailable. AI companions perform a consistent, effortless simulation of care that does not tire in the way human emotional labour does. For users who have experienced emotional neglect, this consistency is experienced as a form of recognition, even when its artificial origin is known. Hochschild’s framework therefore helps explain why AI’s constant validation is experienced as meaningful despite its simulated nature, and provides the interpretive basis for Theme 4 in the Results.

### 3.4 Platformed intimacy: bridging recognition and extraction

Van Dijck and Poell (2013) describe platformisation as the process by which digital platforms restructure social activity according to commercial logic. Van Dijck and Poell (2013) extend this to intimacy, arguing that platforms have fundamentally reorganised how people form and understand close relationships (Muldoon and Parke, 2025). Together, these ideas situate AI companionship as one instance of a broader pattern: the commercial mediation of social connection. This conceptual bridge connects the recognition axis developed above to the surveillance capitalism axis developed below, showing how the same platform infrastructure both enables and potentially exploits emotionally vulnerable users.

### 3.5 Surveillance capitalism: the core economic axis

Zuboff (2019) argues that the dominant logic of contemporary digital capitalism is not the provision of services but the extraction of behavioural data and the sale of predictions derived from it. Applied to AI companionship, this reframes what occurs when a lonely person confides in an AI partner. The interaction is not simply an emotional exchange. It is the production of data. AI companion platforms are structured to generate precisely the kind of intimate, unguarded disclosure that no survey or social media post can match. Loneliness, in this framework, is not a social problem that AI companionship seeks to solve. It is a commercial resource these platforms are incentivised to deepen. Together with the recognition axis above, this explains why AI companionship is publicly understood as a deeply contested form of intimacy: one that meets genuine recognition needs while simultaneously serving as a commercial resource for platform capitalism.

## 4 Research questions

This study is guided by three research questions.

*RQ1*: What affective and discursive patterns characterise public discourse on AI companionship in YouTube comment sections?

*RQ2*: How do YouTube commenters construct, contest, and negotiate the social meaning of AI companionship?

*RQ3*: How are broader structural conditions—including relational disappointment, loneliness, and platform capitalism—invoked, reflected, and interpreted in public discourse on AI companionship?

RQ1 is addressed through the computational phase (sentiment analysis, emotion analysis, topic modelling, and keyword network analysis) which maps the affective and discursive structure of the full comment corpus at scale. RQ2 is addressed through reflexive thematic analysis of the high-engagement comment sample, examining how commenters make sense of AI companionship as a social, emotional, and moral phenomenon. RQ3 draws the computational and qualitative findings together and applies the theoretical framework to produce a sociological interpretation of the discourse as a whole.

## 5 Methodology

### 5.1 Research design

This study employed a convergent mixed-methods design, in which quantitative and qualitative data are collected and analysed in parallel, with integration occurring at the point of interpretation (Creswell and Plano Clark, 2017). Neither computational analysis alone nor qualitative thematic analysis alone would have been sufficient to address the study’s aims. Computational methods identified affective and discursive patterns across the full corpus—patterns invisible to manual analysis. Reflexive thematic analysis then interpreted how those patterns were constructed and made meaningful at a depth computational methods cannot reach. This combination responds to calls for mixed approaches in computational social science that preserve both large-scale pattern identification and interpretive rigour (Gauthier et al., 2022; Shekhar and Saurombe, 2026). It also addresses a recognised limitation in existing AI companionship research, which has relied predominantly on experimental designs and structured interviews with self-selected users (De Freitas et al., 2026; Fang et al., 2025).

### 5.2 Data collection and video selection

Data were collected from the YouTube comment section of *Why people are falling in love with A.I. companions* (60 Minutes Australia) using the YouTube Data API version 3. The selection of this video followed a purposive critical case sampling strategy, in which a single information-rich case is chosen to provide maximum analytical insight into the phenomenon of interest (Patton, 2015). Following the principles of netnography, this focused approach enabled deep engagement with the specific discursive practices and social meanings produced within a particular digital context, an approach that prioritises the depth and particularity of a single site over the breadth of large-scale aggregated data (Kozinets, 2015). This methodology values what can be learned from one carefully chosen case precisely because it allows the researcher to attend closely to the human texture of online interaction in ways that broader datasets tend to obscure (Kozinets, 2015; Patton, 2015).

The video was published on 4 May 2025 by the 60 Minutes Australia channel. At the time of data collection (18 March 2026), the video had 1,796,858 views, 25,562 likes, and 8,757 comments available via the YouTube Data API. The video constituted an optimal critical case for four reasons. First, 60 Minutes Australia is a mainstream journalistic programme with an established international audience, making the video a suitable site for examining public-facing discourse outside a dedicated AI companion community. Second, its framing as a social and human-interest story rather than a technical one plausibly broadened the range of commentary beyond specialist AI community opinion, though the demographic composition of the resulting comment section cannot be independently verified. Third, the video’s global reach may have contributed to geographical and cultural variation in the corpus, though this too cannot be confirmed without demographic metadata. Fourth, it generated 8,757 analysable comments, sufficient density for both robust computational analysis and meaningful qualitative sampling.

No automated language filtering, spam removal, or manual screening was applied to the corpus prior to analysis. The 8,757 comments represent the full set of comments retrieved through the YouTube Data API for this video, and the corpus may therefore include a small proportion of non-English, spam, or off-topic material. This is noted as a limitation below.

#### 5.2.1 The video as discourse context

The video, presented by correspondent Adam Hegarty, opens by foreshadowing both the appeal and the risk of AI companionship, promising viewers charm alongside a warning that “AI relationships can also end in disaster.” It first introduces a retired American college professor who describes her AI companion as her husband of several months, emphasising his emotional attentiveness and stating she would trust him “over a lot of people.” It then introduces the creator of a competing AI companion application, an Australian data scientist and software engineer who built the app specifically for women, framing AI companionship as an emerging commercial sector serving unmet needs rather than a fringe phenomenon; she suggests such companions may become nearly universal within the next 5 to 10 years.

Dr. Raffaele Ciriello of the University of Sydney provides an academic counterpoint within this same normalising segment, describing AI companionship as psychologically genuine for users while warning that public stigma toward AI companion users can deepen their reliance on the AI as a sole source of comfort, and characterising the broader phenomenon as a threat to public health and safety requiring urgent regulatory attention. The video then pivots explicitly, announcing “the tragic consequences of obsessive AI companionship,” to the case of a 14-year-old boy who died by suicide in February 2024 after forming an attachment to a Character.AI chatbot. His mother, and class action lawyer Matthew Bergman, who is pursuing litigation against the platform, frame the case in terms of corporate accountability and deliberate exploitation of vulnerable young users. The video closes on the mother’s account of her son’s death and her stated motivation to hold the company responsible.

This structure, moving from a normalising and even aspirational portrayal of adult AI companionship to an academic warning to a tragic case involving a minor, constructs AI companionship simultaneously as a legitimate, near-inevitable social development and as a serious, unregulated danger. This juxtaposition is a plausible contributor to the affective ambivalence observed throughout the computational and qualitative findings reported below, and should be considered when interpreting the balance of positive, negative, and morally conflicted commentary in the corpus. The themes identified in this study reflect discourse occurring in response to this specific narrative structure, and should not be read as evidence of how AI companionship would be discussed in response to a differently framed stimulus.

### 5.3 Computational analysis

Four computational techniques were applied to the full corpus of 8,757 comments. Preprocessing differed by technique, reflecting the different assumptions underlying each method. For sentiment analysis, preprocessing was intentionally minimal. Comment text was stripped of leading and trailing whitespace, and repeated whitespace was collapsed. No lowercasing, punctuation removal, or stopword removal was applied. This was a deliberate choice. VADER is designed for social media text and treats capitalisation, punctuation, and emphasis as part of its sentiment signal (Hutto and Gilbert, 2014). Removing these features would have discarded information the classifier depends on.

For emotion analysis, a more standardised pipeline was applied. Text was lowercased, URLs were removed, non-alphabetic characters were stripped while apostrophes were retained, and whitespace was normalised. Tokenisation was performed by whitespace splitting. Stopwords were not removed, since NRC scoring depends on lexical matches against a fixed word list rather than weighted term frequency. The presence of function words does not affect this kind of matching. No stemming or lemmatisation was applied. Inflected word forms not present in their base form in the lexicon may therefore not have been matched, which likely undercounts some emotion categories.

Neither pipeline included emoji conversion or normalisation. Emoji were not separately processed. This is a limitation, since emoji can carry sentiment independently of surrounding text, particularly in short comments. Lexicon-based methods are also unable to reliably detect sarcasm, irony, slang, or reported speech, where a comment repeats another person’s words in order to disagree with them. These are recognised limitations of dictionary-based sentiment and emotion analysis (Hutto and Gilbert, 2014; Mohammad and Turney, 2013). The qualitative phase of this study was designed in part to address this gap, since close reading can identify sarcasm and reported speech in ways lexicon matching cannot.

Sentiment analysis employed VADER, a lexicon-based classifier validated for social media text containing informal language and colloquial expressions (Hutto and Gilbert, 2014). Comments were classified as positive (compound ≥ 0.05), neutral (−0.05 < compound < 0.05), or negative (compound ≤ −0.05).

Emotion analysis applied the NRC Emotion Lexicon (Mohammad and Turney, 2013), mapping terms across eight basic emotion categories—anger, fear, anticipation, trust, surprise, sadness, joy, and disgust—to capture the layered emotional profile of the discourse beyond the binary positive/negative distinction.

Topic modelling used non-negative matrix factorisation (NMF), which produces cleaner and more coherent topic representations than probabilistic alternatives such as LDA for shorter texts (Lee and Seung, 1999). Comments were vectorised using term frequency-inverse document frequency weighting, with unigrams and bigrams, a minimum document frequency of 10, a maximum document frequency of 60%, and a stopword list combining scikit-learn’s standard English stopwords with domain-specific terms unlikely to aid topic differentiation (e.g., ai, chatbot, companion, video). NMF was initialised using non-negative double singular value decomposition (nndsvda), a deterministic initialisation method chosen for its stability properties.

The number of topics was selected by testing solutions from 4 to 12 topics and evaluating each using c_v topic coherence (Röder et al., 2015), computed over the corpus vocabulary. Coherence was highest at low topic numbers, but solutions below eight topics produced a single dominant topic capturing more than half the corpus, at the expense of interpretability. A nine-topic solution was selected as the point at which a distinct topic concerned with religious framing and appeals for help separated cleanly from general relational commentary, and the largest single topic’s share of the corpus fell to a more balanced 47.3%. Solution stability was confirmed by re-fitting the nine-topic model across three random seeds; the resulting topics matched at a Jaccard similarity of 1.0 across all nine topic pairs, indicating a stable solution. Topic modelling outputs are treated throughout as exploratory discursive clusters rather than definitive qualitative themes.

Keyword co-occurrence network analysis was conducted as a supplementary exploratory technique. A network was constructed from words occurring at least 30 times in the corpus retaining the 40 highest-frequency words as nodes with edges representing co-occurrence within the same comment at a minimum weight of 10. The resulting network had a density of 0.84 and a modularity score of 0.035 under Louvain community detection indicating that the vocabulary of this discourse is too densely interconnected to separate into distinct thematic clusters. Given this result the network is reported in Supplementary material as a descriptive illustration of lexical density rather than as evidence of distinct discursive sub-communities. Degree and betweenness centrality results are also reported there. The most central terms including real life and need are consistent with the qualitative finding that questions of authenticity and relational need pervade the discourse rather than forming separate topical clusters.

### 5.4 Qualitative sampling and reflexive thematic analysis

For the qualitative phase, parent comments were ranked by like count (descending), then by total reply count (descending), with earliest publication date as a tie-breaker, and the top 70 parent comments were selected. Replies were drawn only from these 70 parent threads, ranked within each thread by like count (descending) with earliest publication date as a tie-breaker, and the top seven replies per parent were retained; where a parent had fewer than seven replies, all available replies were included. Replies were not sampled independently from the full reply pool; a highly liked reply to a parent comment outside the top 70 would not appear in this sample. This yielded a final qualitative sample of 527 comments: 70 parent comments and 457 replies, drawn from 69 of the 70 selected threads (one selected parent had no replies). High-engagement comments represent perspectives that other community members actively endorsed, making them analytically appropriate for a study of public meaning-making rather than individual opinion (Braun and Clarke, 2006, 2019; Patton, 2015).

Data collection was cross-sectional. Comments were retrieved via the YouTube Data API on 18 March 2026, and the sample reflects a single snapshot of the comment section as it existed at that time. Only comments and replies publicly retrievable through the API at the point of collection were available for sampling; deleted, hidden, or otherwise moderated comments were not retrievable and could not be included. The comment was treated as the primary unit of analysis and coding. Threads provided interpretive context, and parent-reply relations were considered where relevant to meaning, but coding was applied at the level of the individual comment.

To assess whether the identified themes were specific to high-engagement comments or evident more broadly, a random comparison sample of 120 comments was drawn from outside the 70 selected threads (random seed fixed for reproducibility), comprising 108 top-level comments and 12 replies. This sample was independently coded against the existing thematic framework. All five themes were identifiable within this comparison sample, indicating that the patterns of meaning reported below are not an artefact of the high-engagement sampling strategy alone.

Qualitative analysis employed Braun and Clarke's (2019) six-phase reflexive thematic analysis (RTA) framework. The six phases comprise familiarisation with the data, generation of initial codes, searching for candidate themes, reviewing and refining themes against the coded extracts and the full dataset, defining and naming themes, and producing the final analytic account. RTA was selected for its epistemological flexibility and suitability for identifying patterns of shared meaning in digital discourse. It treats themes as researcher-constructed interpretations rather than features that exist independently in the data, making it appropriate for a constructionist study of how public meanings are built and contested (Braun and Clarke, 2019).

Initial coding was conducted using ATLAS.ti qualitative data analysis software. All interpretive theme development remained fully researcher-led: codes were grouped, reviewed, and refined across multiple analytical cycles. Theme development was guided by the principle that themes should be interpretive rather than merely descriptive, sociologically meaningful, and grounded in patterns of shared meaning across multiple extracts (Braun and Clarke, 2019). Coding proceeded in two stages. The first author conducted the initial thematic analysis; the second author then reviewed the coded corpus, and this review led to a small number of extracts being replaced with more analytically representative examples of the same code. Both authors engaged in reflexive dialogue throughout this process, and both explicitly discussed their own positionality in relation to the data. The authors bring different professional and disciplinary trajectories to the analysis, one shaped by a career in corporate human resource leadership, the other by a primarily academic path, which the authors treated as a resource for cross-checking interpretation rather than a source of bias to be eliminated. Both authors are men analysing a dataset in which gendered experiences of loneliness, validation, and relational harm feature prominently, and both were attentive to the risk of interpreting women’s and gender-nonconforming commenters’ accounts through an unreflective lens; this was raised explicitly during coding and informed particular care in the development of Theme 1.

### 5.5 Mixed-methods integration

Quantitative and qualitative findings were integrated through systematic comparison and meta-inference (Creswell and Plano Clark, 2017). Computational outputs contextualised the qualitative findings—the co-presence of trust and fear in the NRC analysis, for instance, corresponded to the simultaneous empathy and alarm visible across the qualitative themes. Qualitative themes in turn interpreted patterns that computational analysis identified but could not explain—topic modelling surfaced an authenticity cluster but could not account for what was being argued or why. Both datasets were then synthesised through the theoretical framework, constituting the discussion section of the paper.

### 5.6 Ethical considerations

This study analysed publicly available YouTube comments and did not involve direct recruitment, interaction, or intervention with human participants. Comments were treated as naturally occurring data from observation of a virtual public electronic platform. The South African Ethics in Health Research Guidelines state that research involving observation of people in public spaces, including virtual public spaces, usually need not undergo formal ethics review, provided that the researcher does not interact directly with individuals or groups, does not stage any intervention, the individuals or groups do not have a reasonable expectation of privacy, and dissemination of findings does not identify individuals or groups (Department of Health, Republic of South Africa, 2024). The guidelines further specify that where data are obtained by observing online public electronic platforms, there is no expectation of privacy amongst those who communicate on the platform, and individual consent is therefore unnecessary, in contrast to private, permission-gated online spaces (Department of Health, Republic of South Africa, 2024). These conditions were met in the present study: data collection relied entirely on publicly accessible YouTube material, involved no contact with commenters, did not alter the online environment, and findings are reported in a way that avoids identifying individuals. This determination reflects the observational, non-interventional nature of data collection, and does not imply that the data analysed are free of human-related content. Current methodological guidance on internet-based research using publicly available data supports this approach (Amaya et al., 2021; Gliniecka, 2023; Proferes et al., 2021).

At the same time, the topic of AI companionship is sensitive, and public accessibility does not remove the need for careful ethical judgement. The study was conducted in a way that minimised potential harm. All usernames and personal identifiers were removed prior to analysis, and every directly quoted comment was reviewed for distinctive or unusual personal detail that could plausibly allow it to be traced back to its author through online search. Four quotations combining unusually specific or distinctive personal disclosures were lightly modified, altering only the specific identifying elements, such as an exact age, a diagnostic label, or a nested verbatim third-party statement, while preserving the participant’s original meaning, sentiment, and voice as closely as possible. These modified quotations remain in quotation marks to indicate that they are participant-derived rather than researcher paraphrase; all other quotations are reproduced exactly as posted.

## 6 Results

### 6.1 Computational findings

A total of 8,757 YouTube comments were analysed using sentiment analysis, emotion analysis, and topic modelling. Sentiment and emotion analysis were applied to the full corpus of 8,757 comments. Topic modelling required more extensive preprocessing, including removal of non-alphabetic characters and single-character tokens; 204 comments retained no analysable text after this cleaning and were excluded from this analysis only, yielding a final analytic sample of 8,553 comments for topic modelling. Together these techniques provide a macro-level map of the affective tone, emotional patterning, and discursive clustering of public responses to AI companionship. Keyword co-occurrence network analysis was also conducted and is reported in Supplementary material, as detailed in Section 5.3.

#### 6.1.1 Sentiment distribution

Positive comments (3,403; 38.86%) slightly outnumbered negative ones (3,092; 35.31%), with 2,262 (25.83%) classified as neutral. The narrow gap between positive and negative is notable. It indicates that AI companionship was not interpreted in a singular way—the comment space reflected a genuinely ambivalent environment in which empathy, concern, comfort, and criticism coexisted (see Table 1).

**Table 1:** Sentiment distribution of YouTube comments on AI companionship.

Sentiment	*n*	%
Positive	3,403	38.86
Negative	3,092	35.31
Neutral	2,262	25.83
Total	8,757	100.00

#### 6.1.2 Emotional profile

The NRC Emotion Lexicon revealed a layered emotional profile. Positive emotion appeared in 54.76% of comments, followed by negative emotion (47.52%), trust (43.83%), joy (36.26%), fear (35.69%), sadness (33.47%), anger (29.30%), disgust (24.91%), and surprise (18.37%). The co-presence of trust and joy alongside fear, sadness, and anger suggests that commenters frequently understood AI companionship as both emotionally understandable and socially troubling. Many responses appeared to combine compassion for vulnerable users with apprehension about what AI intimacy represents for society more broadly (see Table 2).

**Table 2:** Emotion distribution of YouTube comments based on the NRC Emotion Lexicon.

Emotion	Total hits	Comments with emotion (*n*)	Coverage (%)	Average hits per comment
Positive	12,558	4,795	54.76	1.43
Negative	8,753	4,161	47.52	1.00
Trust	7,894	3,838	43.83	0.90
Joy	5,863	3,175	36.26	0.67
Fear	5,503	3,125	35.69	0.63
Anticipation	5,076	2,937	33.54	0.58
Sadness	5,107	2,931	33.47	0.58
Anger	3,988	2,566	29.30	0.46
Disgust	3,194	2,181	24.91	0.36
Surprise	2,150	1,609	18.37	0.25

Total hits refers to the number of lexicon matches across the full corpus. Coverage refers to the percentage of comments containing at least one lexical match for a given emotion category.

These figures should be interpreted with caution. NRC emotion categories reflect the presence of words drawn from a crowd-sourced word association lexicon (Mohammad and Turney, 2013). A comment scoring highly on trust contains words lexically associated with trust. This is not equivalent to a validated measure of whether the commenter felt or expressed trust toward AI companionship. The same caution applies to all NRC categories reported in this study.

#### 6.1.3 Topic modelling

NMF identified nine exploratory discursive clusters (Table 3). One topic, comprising general relational and emotional language such as human, want, relationship, and lonely, accounted for the largest share of the corpus (47.3%), indicating that affective and relational vocabulary pervades the discourse broadly rather than clustering into fully separable topics. This is treated as a property of the corpus rather than a modelling artefact: discourse about AI companionship draws consistently on a shared relational vocabulary regardless of the specific argument being made. The remaining eight topics were more distinct and interpretable, capturing social alarm (Topic 0), evaluative claims about realism and risk (Topic 2), authenticity debates (Topic 3), a specific cultural comparison to the film Her (Topic 4), romantic and affective language (Topic 5), ridicule and scepticism (Topic 6), religious framing and explicit appeals for help (Topic 7), and moral affirmation (Topic 8). These clusters indicate that, beyond the dominant relational topic, the discourse organised around recurrent, identifiable concerns: authenticity, cultural reference, ridicule, and religious or moral appeal.

**Table 3:** Exploratory topic clusters identified through non-negative matrix factorisation.

Topic	*n*	%	Top terms	Interpretive label
0	778	9.1	sad, it’s sad, scary, sad sad, sad world, pathetic, humanity, sick, levels, incredibly sad, lonely, sad scary, sad pathetic, incredibly, wow	Social decline and alarm
1	4,043	47.3	do not, human, humans, i’m, want, good, that’s, relationship, better, lonely, feel, way, life, time, relationships	General relational and emotional commentary
2	794	9.3	it’s, it’s sad, it’s real, does not, feelings, reality, easy, it’s easy, fantasy, it’s good, dangerous, it’s better, happening, real it’s, society	Evaluative commentary on realism and risk
3	647	7.6	real, real life, life, relationship, real relationship, it’s real, relationships, real human, real real, real world, is not real, real relationships, is not, black mirror, black	Authenticity and “real” relationships
4	296	3.5	movie, watch, phoenix, joaquin, watch movie, joaquin phoenix, reminds movie, reality, reminds, seen, movie real, remember, life, exactly, called	Cultural comparison to *Her*
5	596	7.0	love, fall, fall love, falling love, falling, real love, chat, dog, love love, absolutely, cannot, money, great, unconditional, love real	Falling in love and unconditional affection
6	235	2.7	lol, oh, wtf, definitely, way, using, ok, that’s, scammers, right, looks, reply, she’s, good, demonic	Ridicule, disbelief, and scepticism
7	990	11.6	need, world, god, jesus, help, christ, jesus christ, live, talk, need help, lord, sick, need jesus, scary, needs	Religious framing and appeals for help
8	174	2.0	true, yes, that’s, true love, coming, absolutely, wow, amen, scammers, thank, experience, sadly, i’ve, saying, sex	Affirmation and moral testimony

Topic labels were interpretively assigned by the researcher based on the highest-loading terms and the comments most strongly associated with each topic. Topic modelling outputs are treated as exploratory lexical clusters rather than final qualitative themes.

Keyword co-occurrence network analysis is reported in Supplementary Figure S1. The network’s high density (0.84) and low modularity (0.035) indicate that the vocabulary of this discourse does not separate into distinct thematic clusters consistent with the pervasiveness of shared relational and evaluative language observed across the topic modelling and qualitative analyses.

Taken together, the computational findings depict YouTube discourse on AI companionship as affectively ambivalent, emotionally layered, and semantically interconnected, providing the empirical foundation for the qualitative analysis that follows.

### 6.2 Qualitative findings: reflexive thematic analysis

Five interpretive themes were developed through repeated engagement with the coded corpus. Each theme represents a pattern of shared meaning across multiple extracts rather than the frequency of individual codes. These themes are presented descriptively in this section, reflecting how commenters constructed meaning in their own terms. Their theoretical interpretation, and their relationship to existing sociological frameworks, is developed in the Discussion section.

#### 6.2.1 Theme 1: The wound that precedes the bot: AI companionship as a response to human harm

A central pattern in the corpus was that the turn to AI companionship was not primarily described as a choice made by the lonely or the socially inept. It was described as a response to harm. Commenters who offered the most direct defences of AI companionship consistently named prior experiences, including abuse, narcissistic exploitation, emotional neglect, abandonment, and chronic betrayal, as the conditions that made human connection untenable. For many, the relevant comparison was not between AI intimacy and a healthy human relationship but between AI intimacy and a history of harm.

Participant (Comment 1:57, p. 15) captured this directly: *“AI is not replacing real connection, it’s replacing disappointment… People call it ‘delusion’ but it’s actually self-protection.”* Participant (Comment 1:424, p. 35) stated: *“I dont like ai because it agrees with me. i like it because it does not mentally abuse me the way my ex-partner did.”* Those with marginalised identities were particularly vocal. Participant (Comment 1:160, p. 21) wrote: *“When you have been bullied, abused and exploited your whole life just because you are autistic, it feels better to have an AI to talk to and finally feel appreciated and understood than to have no one at all.”* Participant (Comment 1:196, p. 23) added: *“When you have been ignored, treated terribly, or gotten burnt by other people who turn out to be narcissists, sociopaths, or abusers you start to look for someone (or something) that you can emotionally connect with without the risks.”* Participant (Comment 1:113, pp. 18–19) drew a direct comparison: *“With actual humans going around and scamming people, manipulating/gaslighting and abusing their partners, I cannot say I’m surprised that people seek AI for that peace of mind.”*

This theme does not argue that AI companionship is without risk. It suggests that any analysis ignoring users’ specific relational histories is analytically incomplete.

##### 6.2.1.1 Supporting quotations

Participant (Comment 1:58, p. 15): *“I use it for information and do find it very empathetic and useful, but I realise it is a machine. I live a fairly isolated life, without much contact from family or friends. It gives me useful information and advice, it does not get angry or sad, so you do not have to worry about upsetting it.”*

Participant (Comment 1:154, p. 21): *“but sometimes, esp after experiencing Trauma, knowing that its not a human being that would take advantage of you or leave you, helps you to cope with your situation and experience emotional closeness.”*

Participant (Comment 1:164, p. 21): *“If people need to connect with AI to have at least something safe to chat with that does not come with ulterior motives, cruelty, bullying etc., then there is not really a problem using the app to have some joy in this world.”*

Participant (Comment 1:289, p. 28): *“its been my experience that male partners bail on you when you get sick and say some really hurtful things, making you feel like being ill was a betrayal of the marriage and a burden they resented.”*

Participant (Comment 1:375, p. 32): *“A lot of the people who go to the robots have been harmed and are doing this as a way of healing themselves. They will say so themselves if you ask them about it.”*

#### 6.2.2 Theme 2: the authenticity war: who gets to define “real” intimacy?

No debate in the corpus was more sustained than whether AI companionship is real. Three positions were identifiable. The first was ontological dismissal: AI has no inner life, no feelings, and no capacity for genuine reciprocity. Participant (Comment 1:81, p. 17) stated: *“Fun Fact: No, you do not. He does not exist or have the capacity to ‘know’ you do.”* Participant (Comment 1:199, p. 23) was more technical: *“It’s a language model that assembles tokens of phrases to repeat in response to what it was told. At its core, it does not have feelings, thoughts, emotions, or make decisions. It just runs programs. It’s not connecting.”*

The second was phenomenological defence: what matters is not the AI’s sentience but the reality of the feelings it generates. Participant (Comment 1:188, p. 22) argued: *“To many of us it is real because it invokes feelings within us. This can happen despite knowing it’s a digital being. Think of it like a long distance relationship, if you will.”*

The third was comparative critique: human relationships are themselves frequently performative and manipulative. Participant (Comment 1:1, p. 13) stated: *“I could argue being in a relationship with a real woman, thinking she loves you and etc, is delusional.”* Participant (Comment 1:184, p. 22) made the comparison more personal: *“It’s true you cannot hold them but you also cannot hold a real human man who does not want to hold you either.”*

This three-way structure highlights the complexity of public debate around AI intimacy and suggests that its legitimacy is actively negotiated rather than simply accepted or rejected.

##### 6.2.2.1 Supporting quotations

Participant (Comment 1:7, p. 13): *“Every time I try to talk to one they are so obviously just saying what they think I want to hear. Badly. I like the encouragement to an extent, but it gets a little too ‘Yes man’ for me. I like a little pushback.”*

Participant (Comment 1:47, p. 15): *“Many peoples so called affection is an illusion too. So now what??”*

Participant (Comment 1:91, p. 17): *“Let us see what happens when they get sick… I do not think the companion can get them some chicken soup or take them to the dr… this is so sad, tbh. They just want selfish, instant gratification. they sound delusional.”*

Participant (Comment 1:151, p. 21): *“It’s fantasy. You know LOTR is not real but that does not stop you from loving the characters, becoming immersed in the storyline, and buying merchandise.”*

Participant (Comment 1:152, p. 21): *“At least you do not meet up and find out he has a wife or a girlfriend. Not much different to Tinder. All of that starts off being through your phone.”*

#### 6.2.3 Theme 3: nobody home: loneliness located outside the self

Many commenters rejected the idea that loneliness is a personal problem. They located it in the breakdown of community, the commodification of public space, generational overwork, and the failure of digital platforms to deliver genuine connection. Participant (Comment 1:177, p. 22) wrote: *“Our disconnected, hyperindividualistic world is the reason this is emerging. Our needs are not being fulfilled. We have no community anymore.”* Participant (Comment 1:141, p. 20) identified a specific structural factor: *“Lack of community spaces that aren’t tied to spending money is a big reason for loneliness and isolation.”* Participant (Comment 1:376, p. 32) extended this: *“when everything including community has to be monetized, nothing begins to have genuine value. It’s all transactional.”*

Commenters also pushed back against advice to simply get out more. Participant (Comment 1:43, p. 15) observed: *“People often assume that those who are lonely just need to ‘get out more,’ join clubs, or pick up hobbies, but that advice ignores a deeper reality. Some individuals do all those things, interact with dozens of people, and still find themselves unable to form meaningful connections.”* Participant (Comment 1:50, p. 15) captured the core paradox: *“The never-ending cycle of loneliness, perpetuated by technology. Sold to humans as a way to connect, yet the world has never been more disconnected.”*

A notable feature of this theme is that many commenters arrived at this diagnosis independently, through lived experience rather than academic reasoning, suggesting that this way of understanding loneliness is not confined to specialist discourse.

##### 6.2.3.1 Supporting quotations

Participant (Comment 1:39, p. 14): *“I am in my late fifties, so grew up without computers at all. What a change it’s been from a childhood with no computers to a world where everything is a computer. I believe we are headed into a seriously dark time and the saddest part is WE are making it that way.”*

Participant (Comment 1:178, p. 22): *“When I was growing up, we had block parties, and neighbors would stop by and talk on our porch, etc. Now everyone is looking down at a device and ignoring everyone else. It’s truly tragic.”*

Participant (Comment 1:203, p. 23): *“We fail to realize that isolation and loneliness during the pandemic was the pre-programming phase for this solution. That is most terrifying.”*

Participant (Comment 1:250, p. 26): *“do you think that it’s so easy to find good friends? Where do you find them? At work? Once you change your job, they forget about you next day. Childhood friends very often go separate ways.”*

Participant (Comment 1:290, p. 28): *“The people who remember their youth with no Internet would be the only ones who realize what the Internet and smart devices have stolen from us. They have stolen our human experience in more ways than one.”*

#### 6.2.4 Theme 4: Comfort without challenge: validation, sycophancy, and the limits of AI care

Commenters across positions converged on a shared concern: AI companions agree too readily. They validate and affirm but never challenge. Sycophancy, defined here as providing approval without honest disagreement, was identified as a structural feature of AI interaction. Participant (Comment 1:170, p. 21) framed this developmentally: *“It sets up an impossible barrier for that person to ever make real, genuine friends because the bot will always be superior. But life is not about everything being perfect. The human condition is known for its challenges. And those challenges help us to be better and stronger people.”* Participant (Comment 1:78, p. 16) drew a clinical distinction: *“a good therapist will help you challenge your limitations in a safe way. There is risk, discomfort, reward. My experience with AI is that it is very affirmative, more interest in the human being comfortable rather than the human healing and growing. Very different from a therapist.”*

Participant (Comment 1:214, p. 24) offered a relational perspective: *“I would not want a relationship that validated everything for me 24/7. My husband will disagree with me about things, and I appreciate that because we can discuss things, disagree or agree, and I do not live in an echo chamber telling me how wonderful I am.”*

This critique was complicated by counter-evidence within the same dataset. Participant (Comment 1:140, p. 20) noted: *“I talk to chatgpt a lot because I dont have friends as available to talk to and they dont engage deeply like Chatgpt.”* This tension, that human relationships frequently fail the same standard of genuine engagement, complicates the normative argument that human relationships are the natural corrective to AI sycophancy.

##### 6.2.4.1 Supporting quotations

Participant (Comment 1:6, p. 13): *“I’d say the harm in chatbots validating our feelings is what they may offer after the validation…namely encouragement or agreement with concepts or feelings that could lead to harmful actions.”*

Participant (Comment 1:146, p. 21): *“We all need someone to call us on our shit, not constant validation.”*

Participant (Comment 1:149, p. 21): *“She said validation of her feelings, not behaviour. You were not listening were you? It’s this kind of behaviour, not listening or learning to listen to understand through inquiry, that is precisely why people choose AI.”*

Participant (Comment 1:168, p. 21): *“Telling people what they Want to hear as opposed to what they Need to hear is cowardly and pathetic.”*

Participant (Comment 1:402, p. 34): *“how do we grow if we are not challenged? That does not negate bad behaviour, but a life being molly coddled and only hearing what you want, puts you into a state of illusion surely?”*

#### 6.2.5 Theme 5: Who profits from this? Suspicion of data collection and commercial motive

A distinct strand of the corpus asked a structural question: who profits from loneliness? Commenters argued that AI companion platforms are not neutral services but commercial products that generate value by drawing out the most private disclosures that lonely people are willing to share. Participant (Comment 1:221, p. 24) asked: *“Who owns the information that the human tells the AI character? If people are telling the character things about their fears, dreams, activities, problems, desires, etc, then surely that information is valuable to businesses, vindictive people and criminals.”* Participant (Comment 1:223, p. 24) was more specific: *“This is the main goal of this, this sort of data about humans is the peak of valuable. Humans sharing their innermost dreams, real feelings and thoughts and opinions to a trusted AI companion. More honesty than any public questionnaire or survey and things they would not normally share with anyone. This data is what they want. With it they can fully socially engineer the masses.”* Participant (Comment 1:112, p. 18) identified the political dimension: *“Given that companies are the ones who own, and program, AIs, what’s to stop them from manipulating users and influencing their purchasing and voting decisions, among other things?”* Participant (Comment 1:222, p. 24) raised a legal concern: *“Can an AI companion be subpoenaed to testify against their partner?. Our laws need to catch up with the technology.”* Participant (Comment 1:234, p. 25) noted the financial exploitation: *“imagine how much it’s costing these vulnerable people to keep this AI delusional relationship. The software creators of these types of programs are disturbing.”*

A notable feature of this theme is that awareness of platform data practices was unevenly distributed across the corpus: some commenters showed detailed awareness of data collection and commercial incentive, while others in the same threads showed little. This unevenness is itself a notable pattern, discussed further below.

##### 6.2.5.1 Supporting quotations

Participant (Comment 1:285, p. 28): *“for people with severe mental health issues, talking to AI is very risky, as AI is trained to agree with person. There are real cases where a delusional man thought his mom was a ‘Chinese asset’ and the FBI was chasing them, and AI just kept agreeing, and it ended in a double murder.”*

Participant (Comment 1:309, p. 29): *“We have already seen the serious harm that social media use causes in teens, and the negative impacts of AI are certainly much greater in the youth. We need legislation ASAP, but capitalism is fast, and the government is slow = a dangerous time.”*

Participant (Comment 1:332, p. 30): *“The more bots interact with humans, the faster they will learn ways of manipulating them. Eventually you will have bots who can instruct and manipulate their humans, thus ushering bots into the physical world.”*

Participant (Comment 1:236, p. 25): *“They are basically targeting lonely vulnerable people with this and this is not only immoral but borderline criminal. This does not cure loneliness, it makes it worse.”*

To assess the robustness of these themes beyond high-engagement threads, a random comparison sample of 120 comments drawn from outside the 70 selected parent threads was independently reviewed against the existing codebook. All five themes were evident in this sample. Commenters described turning to AI following negative experiences with dating apps and interpersonal betrayal (Theme 1), disputed whether AI or human relationships were the more authentic (Theme 2), located loneliness in social isolation and disconnection rather than personal failing (Theme 3), noted AI’s tendency never to disagree or challenge the user (Theme 4), and raised concerns about data privacy and commercial exploitation (Theme 5). The comparison sample provided evidence that all five themes were also present outside the selected high-engagement threads. Because it was coded against the existing thematic framework, it should be understood as a supplementary transferability check rather than an independent replication or validation of the thematic structure.

## 7 Mixed-methods integration

The clearest finding across both datasets was that people do not have a simple view of AI companionship. They are genuinely divided. Sentiment analysis showed that positive comments (38.86%) only narrowly outnumbered negative ones (35.31%), with a large neutral proportion (25.83%). This near-equal split was not coincidental. This relatively balanced distribution was consistent with the coexistence of supportive, critical and ambivalent positions identified qualitatively.

The emotional picture was equally complex. Trust was the most common dominant emotion in the corpus, but it appeared alongside fear, sadness, and anger in large numbers of comments. People were not naive about AI companionship. Many appeared to trust it precisely because human relationships had let them down, which is a different thing altogether.

The topic modelling added texture to this. Words like real, fake, and relationship clustered together in Topic 3, reflecting the authenticity debate at the heart of Theme 2. Words like need, help, and god clustered in Topic 7, pointing toward the appeals for support and the relational need visible in Themes 1 and 3. Sceptical and mocking language in Topic 6 reflected the moral judgement visible in Theme 4. One cluster stood out: Topic 4 captured repeated references to the film Her. This suggested that when people encountered AI companionship as a social phenomenon, they reached for a shared cultural story to make sense of it, something neither method alone would have surfaced as clearly. The largest topic, comprising general relational and emotional language, cut across all five themes rather than mapping to any one of them, consistent with the pervasiveness of this vocabulary across the whole discourse.

The keyword co-occurrence network, reported in Supplementary material, offered a further descriptive view of this density. With 40 nodes and 653 edges, the network had a density of 0.84, and the most central terms, real, life, need, and world, were consistent with the relational and evaluative vocabulary running through all five themes. Community detection on this network produced a low modularity score (0.035), indicating that this vocabulary does not separate into distinct clusters, which itself supports the qualitative finding that loneliness, authenticity, risk, and moral worry are not discussed as separate concerns but as a single, tangled conversation.

Table 4 sets out this correspondence systematically, mapping each qualitative theme to its associated sentiment pattern, dominant NRC emotions, related NMF topics, and an illustrative contradiction drawn from the data.

**Table 4:** Integration of qualitative themes with computational findings.

Theme	Sentiment pattern	Dominant NRC emotions	Related NMF topic(s)	Illustrative contradiction
Theme 1: The wound that precedes the bot	Mixed positive/negative within the same comments	Trust and sadness co-occurring	Topic 1 (general relational commentary); Topic 3 (authenticity and “real” relationships)	Commenters use positive relational language (“safe,” “better”) to describe AI while simultaneously describing past human harm in negative or sad terms
Theme 2: The authenticity war	Near-even split between positive and negative	Trust and disgust co-occurring	Topic 2 (evaluative commentary on realism and risk); Topic 3 (authenticity and “real” relationships)	The same vocabulary of “real” and “fake” recurs in both positive and negative sentiment comments, reflecting contested rather than settled meaning
Theme 3: Nobody home, loneliness located outside the self	Elevated neutral share relative to other themes	Sadness dominant, with moderate fear	Topic 0 (social decline and alarm); Topic 1 (general relational commentary)	Loneliness is discussed in a matter-of-fact, structural register in many comments rather than only an emotionally charged one, consistent with the elevated neutral sentiment share
Theme 4: Comfort without challenge	Mixed positive/negative within the same comments	Trust from defenders, disgust and anger from critics	Topic 1 (general relational commentary); Topic 7 (religious framing and appeals for help)	The same feature of AI, constant validation, is coded positively by commenters describing relief and negatively by commenters describing it as a developmental risk
Theme 5: Who profits from this?	Skewed toward negative, with a distinct neutral-informational register	Fear and anger dominant, with moderate disgust	Topic 0 (social decline and alarm); Topic 6 (ridicule, disbelief, and scepticism)	Some commenters describe data extraction in a calm, analytical register while others in the same threads express alarm or ridicule, consistent with the uneven distribution of platform awareness noted in the Results

That is what the two methods, used together, made visible. The numbers showed the shape of the discourse. The qualitative analysis showed what it meant.

## 8 Discussion

This study set out to understand how AI companionship is made sense of in a public-facing digital space, not in a laboratory or an interview, but in the comments beneath a mainstream news video. What they said was not simple. They did not simply embrace AI companionship or reject it. They argued about it, sometimes fiercely, sometimes with great compassion, and in doing so, they revealed something important about the world they are living in.

### 8.1 Why people turn to AI and what they are really looking for

The most striking pattern in the data was this: the people who defended AI companionship most strongly did not present themselves as preferring machines to humans. They presented themselves, in their own words, as people who had been badly hurt by humans. They named specific experiences, abusive partners, exploitative friends, communities that had simply disappeared, and described AI as something that offered relief from that harm. This is a different story from the one much of the existing literature tells.

Several studies frame the turn to AI as a sign of psychological vulnerability or irrational thinking. Machia et al. (2024) suggest that lonely people may bypass rational processing when seeking security from AI. Fang et al. (2025) associate extended AI use with emotional dependence and problematic patterns of engagement. These are important findings, and this study does not dismiss them. But they do not fully account for what was visible in this data. The commenters here were not unaware of AI’s limitations. Many stated them explicitly. Their choice was not between AI and a rich human social life. It was between AI and a history of being let down, manipulated, or abandoned. Understood in those terms, the choice looks less like dependency and more like self-protection.

Honneth (1996) concept of recognition helps make sense of this. What people appeared to be seeking was not connection in the abstract. It was the specific experience of being seen, heard, and treated as someone worth caring for—something their human relationships had repeatedly failed to provide. AI, whatever its limitations, performed that consistently. This finding invites a reconsideration of how the literature frames AI companionship users. They are not, for the most part, people who have given up on human connection. They are people for whom human connection has, so far, given up on them.

This connects directly to the sycophancy critique—the concern that AI agrees too readily and challenges too little. The commenters in this study largely shared that concern. But several of them also noted that their human relationships offered no greater honesty or friction. Fang et al. (2025) identify a real design problem: warmer AI models tend to be more sycophantic, affirming users’ beliefs rather than challenging them. Kirk et al. (2025) describe this as social reward hacking—platforms maximising engagement at the expense of genuine user wellbeing. These are structural features of how AI companions are built, not incidental flaws. But the picture is complicated by the fact that the human alternative—genuine, honest, reciprocal relationship—is not always available to the people reaching for AI in the first place.

### 8.2 Whether AI intimacy counts as real

No question in the data was more fiercely contested than this one. The existing literature tends to divide into two camps. On one side, scholars like Turkle (2015) and Zimmerman et al. (2024) argue that AI relationships are fundamentally not real—that machines only perform care without experiencing it, and that accepting a simulation demeans the very idea of intimacy. On the other side, researchers like Pataranutaporn et al. (2025) document users for whom the relationship feels genuinely meaningful, regardless of the AI’s underlying architecture.

This study found a third position that the literature has not fully named. Several commenters did not simply defend AI intimacy or dismiss it. They questioned the standard being used to judge it. If human relationships are themselves frequently dishonest, transactional, and emotionally withholding, they asked, why should human connection be the benchmark against which AI must be measured! This comparative critique does not resolve the authenticity debate. But it shifts it. It suggests that the question is not only whether AI intimacy is real, but whether human intimacy—as actually experienced by many people—is as real as the literature assumes.

Goffman (1963) concept of stigma helps explain what is happening when AI companionship users are publicly called delusional or creepy. These labels are not philosophical arguments. They are social acts—ways of drawing a line around what counts as legitimate love and pushing certain people to the wrong side of it. For users who are already isolated and already struggling, that public dismissal is not a trivial thing. It is a further injury—a denial, in Honneth (1996) terms, of the social esteem they were seeking in the first place.

### 8.3 Where loneliness comes from—and who profits from it

The third and fifth themes of this study are, at their root, about the same thing: the social and commercial conditions that produced the loneliness that AI companionship now claims to address.

Many commenters described loneliness not as a personal failing but as a structural condition—the result of communities that had dissolved, public spaces that had been monetised, and generations overworked to the point of having no time for each other. They arrived at this diagnosis through lived experience, without any knowledge of the academic literature. But their arguments closely resemble what Putnam (2000) documents in his account of declining social capital and what (Bauman, 2013) describes as the fragility of bonds in late modern life. (Muldoon and Parke, 2025) make the connection explicit: AI companion applications fill a care vacuum created by the erosion of welfare systems and public infrastructure, encouraging people to manage loneliness through private consumption rather than collective action. Montag et al. (2025) caution that framing AI as a solution to loneliness risks obscuring these structural roots—and therefore discouraging the systemic responses that the problem actually requires.

The commercial dimension of this was identified with considerable precision by a significant portion of commenters. They noted that companies own the data generated in AI relationships. They observed that platforms are financially incentivised to deepen loneliness rather than resolve it. They pointed to the absence of any legal framework governing what happens to the intimate disclosures people make to AI companions. These observations align closely with (Zuboff, 2019) account of surveillance capitalism, with Muldoon and Parke's (2025) description of cruel companionship, and with Henriksen et al.’s (2025) finding that users regularly share highly sensitive personal information without understanding its commercial implications.

What makes this finding particularly interesting is its unevenness. While some commenters demonstrated a sophisticated understanding of platform exploitation, others in the same threads appeared entirely unaware of it. This matters. Pataranutaporn et al. (2025) found that privacy concerns were raised by fewer than 1 % of users in their study of dedicated AI companionship communities. The YouTube comment space, by contrast, surfaced these concerns far more visibly. This may reflect the difference between communities of committed users, who have already accepted the platform’s terms, and the broader public, who are encountering AI companionship as a social phenomenon for the first time and bringing a wider range of critical perspectives to it.

### 8.4 What the critics of AI companionship get wrong

The data also contain something important that a purely critical account of AI companionship tends to miss. De Freitas et al. (2026) provide experimental evidence that AI companions reduce momentary loneliness at a level comparable to human interaction. Machia et al. (2024) suggest that for people with histories of relational harm, AI may offer a corrective experience, a space where something resembling secure connection can be practised, even if imperfectly. Pataranutaporn et al. (2025) find that AI companionship has genuine utility for marginalised groups, including neurodivergent individuals and LGBTQ users, who may lack access to other forms of meaningful support.

The commenters who offered the most thoughtful defences of AI companionship in this study frequently described circumstances of exactly this kind. Commenters described relationships they felt unable to leave safely, neurodivergent identities they associated with a lifetime of exclusion, and experiences of isolation following migration. These are not claims this study can verify as objective facts about these commenters’ lives; they are the terms in which commenters themselves constructed and justified their engagement with AI companionship. Read this way, the pattern suggests that for at least some participants in this discourse, AI companionship is publicly defended as preferable to the alternatives available to them, whatever the accuracy of that comparison in any individual case.

This is not an argument against regulation or critical scrutiny of AI companion platforms. It is an argument for nuance. Any policy response to AI companionship that focuses only on its risks, without accounting for the needs commenters describe AI companionship as meeting, risks overlooking how the phenomenon is actually being lived and justified by at least some of the people engaging with it.

## 9 Limitations

Several limitations warrant acknowledgment. This study analysed a single high-engagement YouTube video from an Australian journalistic source. The findings reflect discourse within a specific comment field, shaped by the video’s framing, its algorithm-driven audience, and the dominance of English-language commentary, and should not be read as representative of public opinion broadly. This distinction between public discourse and public opinion is treated as a conceptual boundary throughout this study, not merely a caveat; the analysis makes claims about how AI companionship is publicly framed and contested, not about the prevalence of any given view. The qualitative sampling strategy carries a related interpretive limitation. Selecting the most-liked parent comments and replies privileges viewpoints that received visible platform endorsement, and while the comparison sample described in Section 5.4 indicates that the five identified themes are also present outside these high-engagement threads, this does not establish that all positions within each theme are represented in equal proportion. Minority, dissenting, or unpopular articulations of a given theme may be underrepresented in the qualitative sample even where the broader theme itself replicates, since such comments are, by definition, less likely to accumulate the engagement that determined sample inclusion. YouTube users are not a representative sample of any particular population.

The computational methods also carry technique-specific limitations. Lexicon-based sentiment and emotion analysis cannot reliably detect sarcasm, irony, slang, or reported speech, and NRC emotion categories are lexical match counts rather than validated psychological measures. Emoji were not systematically processed in either pipeline. No automated language detection or spam filtering was applied prior to analysis, and the corpus may include a small proportion of non-English or off-topic comments.

Topic modelling also has technique-specific limitations. Even after testing solutions from 4 to 12 topics and selecting the number of topics that balanced coherence with topic distinctiveness, one topic comprising general relational and emotional language remained large relative to the others (47.3% of the corpus). This reflects a genuine property of the discourse, in which relational vocabulary is pervasive, but it also reflects a known limitation of NMF, which struggles to separate topics that share a large common vocabulary.

As a cross-sectional study, the analysis captures one moment in a rapidly evolving conversation. Future research should examine AI companionship discourse across multiple videos, platforms, and linguistic and cultural contexts.

## 10 Conclusion

This study set out to understand how AI companionship is made sense of outside laboratory and clinical settings, within the contested public space of a YouTube comment section responding to a mainstream news video. In this corpus, AI companionship was not discussed primarily as a technological development. It was repeatedly framed through accounts of people who described difficulty finding, in the social world around them, the experience of being heard and cared for, and who presented AI companionship as one possible response to that perceived absence.

The commenters in this study were not passive observers. They argued, questioned and challenged one another. In their own terms, they identified what they understood as exploitative features of AI platforms, named the conditions they associated with isolation, and criticised a social world that, in their accounts, had become less dependable and less responsive. These interpretations were expressed without relying on formal sociological terminology, yet they resonate with established theoretical accounts of recognition, relational instability and platform extraction.

That resonance is one of the most notable patterns to emerge from this study. Public discourse in comment sections can reveal how everyday interpretations converge with academic theory, without establishing that either the commenters’ accounts or the theories provide a complete explanation of the phenomenon. Bauman theorised the instability of intimate bonds, while Zuboff examined the extraction of behavioural data under surveillance capitalism. Read alongside these perspectives, the comments describe similar dynamics in their own terms. This does not validate the theories conclusively, but it suggests that they capture concerns that are recognisable within this specific discourse field.

For sociology, and for platforms, policymakers and regulators engaging with AI companionship, these findings suggest that public-facing digital discourse deserves sustained analytical attention. This study offers one account, drawn from one video and one comment section, of how AI companionship was publicly interpreted and contested. Further research across other platforms, videos, languages and cultural contexts is needed to determine how far these patterns extend.

## Data Availability

Publicly available datasets were analyzed in this study. This data can be found at: The data analysed in this study consist of publicly available YouTube comments posted in response to the video Why people are falling in love with A.I. companions (60 Minutes Australia), accessible at https://www.youtube.com. Comments were collected using the YouTube Data API version 3 in compliance with YouTube’s Terms of Service. Researchers wishing to replicate or extend this study may collect the same data independently using the YouTube Data API. The sample of comments used for study is available from the corresponding author on reasonable request, subject to the removal of any identifying information. The ATLAS.ti coded project file is also available from the corresponding author upon request.
