# Supplementary material for: Can’t help falling in love… with a bot? A mixed-methods analysis of public discourses of AI intimacy and companionship on YouTube
Source: Front Sociol. 2026 Aug 5;11:1846117. doi: 10.3389/fsoc.2026.1846117 (PMC13485705; doi:10.3389/fsoc.2026.1846117)
Supplement: Supplementary file 1 [file Supplementary_file_1.docx]

Supplementary figure S1: Keyword Co-occurrence Network of Public Discourse on AI Companionship

Note. The network was generated from high-frequency co-occurring words in the comment corpus. The final filtered network contained 40 nodes and 653 edges.
